# Altered cardiac and vascular stiffness in pregnancy after a hypertensive pregnancy

**DOI:** 10.1038/s41371-022-00662-4

**Published:** 2022-02-25

**Authors:** James S. Castleman, Alena Shantsila, Richard A. Brown, Eduard Shantsila, Gregory Y. H. Lip

**Affiliations:** 1 West Midlands Fetal Medicine Centre, Birmingham Women’s and Children’s Hospital NHS Foundation Trust, Birmingham, UK; 2 University of Birmingham Institute of Cardiovascular Sciences, City Hospital, Birmingham, UK; 3 Liverpool Centre for Cardiovascular Science, University of Liverpool and Liverpool Heart & Chest Hospital, Liverpool, UK; 4 Northumbria Healthcare NHS Foundation Trust, Newcastle upon Tyne, UK; 5 Department of Primary Care and Mental Health, University of Liverpool, Liverpool, UK; 6 Aalborg Thrombosis Research Unit, Department of Clinical Medicine, Aalborg University, Aalborg, Denmark

**Keywords:** Medical imaging, Medical research

## Abstract

Hypertensive disorders of pregnancy are an important cause of morbidity and mortality, impacting on both maternal and fetal wellbeing. Affected women are at higher risk of future cardiovascular morbidity and mortality. Our study objective was to assess differences in cardiovascular function in pregnant women previously affected by gestational hypertension or preeclampsia. Pregnant women diagnosed with gestational hypertension or preeclampsia in a previous pregnancy were recruited at the start of a subsequent pregnancy and compared to healthy pregnant and non-pregnant controls. All patients underwent pulse wave analysis and echocardiography. Indexes of echocardiography-derived arterial and left ventricular elastance were calculated. In our study women with prior hypertension (*n* = 25) were more likely to have blood pressure in the 120–139/80–99 mmHg (prehypertension) range. Women with previous hypertension in pregnancy had increased late diastolic transmitral flow velocities (A wave) and increased augmentation index. Women without prior hypertension (*n* = 50) demonstrated more compliance (reduced EaI and Ees) compared to the non-pregnant controls (*n* = 40). This adaptation was not seen in pregnancy with prior hypertension, where increased arterial stiffness was observed. In conclusion we have shown increased prevalence of prehypertension and increased arterial stiffness in pregnant women previously affected by gestational hypertensive disease. An increased atrial component to ventricular filling reflects altered diastolic function after hypertensive pregnancy. These women are at increased future cardiovascular risk due to altered cardiac and vascular function and require effective risk mitigation.

## Introduction

Hypertensive disorders of pregnancy (HDP) are an important cause of morbidity and mortality, impacting on both maternal and fetal wellbeing. The definition of hypertension in pregnancy requires either a systolic blood pressure (BP) of at least 140 mmHg or a diastolic BP (DBP) of at least 90 mmHg, with a second confirmatory reading separated in time usually by 4 h [1]. BP should be measured with a device validated in pregnancy [2]. Significant proteinuria (urine protein/creatinine ratio of at least 30 mg/mmol [3]) has traditionally been the second criterion required to distinguish gestational hypertension (GH) from preeclampsia (PE). The International Society for the Study of Hypertension in Pregnancy [4, 5] describe PE as a syndrome comprising hypertension and end organ dysfunction, with renal, hepatic, haematological, neurological or placental manifestations. Various maternal and fetal sequelae of the disease now appear in international guidelines for the diagnosis of PE, with proteinuria no longer mandatory [6–9]. A woman affected by PE is at higher risk of future cardiovascular morbidity and mortality [10, 11]. This may be due to persistent changes in cardiac structure and function, or to irreversible injury to the cardiovascular system [12, 13]. We have described echocardiographic cardiac structure and function in HDP in a systematic review [14].

Arterial stiffness, or elastance, defines the change in pressure (∆P, stress) relative to a change in volume (∆V, strain) of blood flow through an artery [15]. Increased vascular and left ventricular (LV) stiffening may lead to an alteration in ventriculo–arterial coupling, which can be assessed reproducibly by echocardiographic measurement of arterial and cardiac elastance [16–19]. A recent systematic review and meta-analysis has demonstrated a significant increase in arterial stiffness indices in women with PE compared to women with GH and normotensive pregnant women [20]. Despite the growing evidence of abnormal arterial stiffness and diastolic dysfunction in pregnancy, the mechanisms of these changes and mutual relationship between arterial and cardiac abnormalities are not clear [21].

We aimed to investigate how maternal cardiac structure and function is affected by a previous hypertensive pregnancy. Ultrasound is currently used to perform a first trimester risk assessment for the fetus but its role in maternal cardiovascular risk assessment is not yet defined. We hypothesised that a history of gestational hypertensive disease would be associated with abnormal ventricular-arterial interaction in a subsequent pregnancy, with reduced arterial elastance and altered ventricular elastance reflecting maladaptation to the pregnant state.

## Methods

“Evaluating Cardiovascular Changes in Hypertension in Obstetrics” (ECCHO) was a prospective observational study in which women were recruited at the beginning of pregnancy and studied throughout their gestation. Cross-sectional comparison in the first trimester of pregnancy tested the hypothesis that prior hypertension in pregnancy is associated with altered arterial and ventricular function. Pregnant women with prior HDP were compared to pregnant women without prior hypertension and to healthy non-pregnant controls.

### Study groups

Three study groups were recruited. Group 1 comprised pregnant women with previous HDP (based on the National Institute of Health and Care Excellence guidance [3]). Group 2 comprised pregnant women with no history of hypertension. Group 3 included healthy non-pregnant women as controls. Patients were recruited from women attending the Department of Maternity and Perinatal Medicine at Sandwell and West Birmingham Hospitals NHS Trust. Eligible women were identified from referrals for antenatal care and approached during their first hospital visit. Non-pregnant controls were recruited from hospital and university staff. A comprehensive medical history was taken from each woman, to assess them against the inclusion and exclusion criteria and to provide the necessary data for the study. Hospital case notes were also cross-examined to confirm the past medical history in order to reduce recall error and bias. Gestational age was determined by fetal biometry at 11–14 weeks. The pregnant women were followed throughout pregnancy and the pregnancy outcome recorded. There were no changes in the routine antenatal care of patients. Clinical management of pregnancy was in accordance with established local protocols, based on national guidelines. Exclusion criteria were pre-existing cardiac disease (ischaemic heart disease, valvular heart disease, congenital heart defect), chronic hypertension, significant co-morbidities, use of vasoactive medication, multiple pregnancy, inability to consent (language barrier with no translator available, lacks capacity), obstetric emergency (haemorrhage, severe symptomatic (pre)eclampsia, presentation in labour) and age under 16 years.

### Echocardiography

Echocardiography was performed using a Philips iE33 ultrasound machine (Bothell, WA, USA) with a phased array transducer. The images were converted to Digital Images and Communications in Medicine format. Xcelera software (Philips Medical Systems, Netherlands) was used to analyse the stored images. After a period of at least 5 min rest, the women were examined in a comfortable left lateral position on the couch. A left lateral tilt was employed throughout the longitudinal study for standardisation. Appointments were routinely made in the morning. Two investigators (AS and RAB), experienced in cardiac imaging and accredited with the British Society of Echocardiography, performed the transthoracic echocardiograms throughout the study. The examination protocol was in accordance with the latest published guidelines from the international societies [22–24].

The images were anonymised and digitally stored prior to offline analysis by a single observer (JSC). All measurements were performed after completion of the study in a random order, with the investigator blinded to the identity of the patient, their clinical characteristics, including BP, and their pregnancy outcome. Measurements from 2D structural images were recorded once. Measurements based on flow/waveforms were performed on four beats and the mean was taken. Measurements of the parameters of arterial-vascular interactions have been previously validated and were done in accordance with established protocols [17].

### Blood pressure measurement

BP was measured with a digital BP monitor (Omron Corporation, Tokyo, Japan). This automated, electronic, oscillometric device is validated for use in pregnancy [2] and was calibrated throughout the study. The brachial artery of the non-dominant arm was used for the BP recording. Care was taken to ensure that the arm was free of clothing and that appropriate cuff size was used depending on mid-arm circumference. BP was recorded whilst the patient was seated comfortably and silently, with the arm supported at the level of the heart. The woman was asked to sit upright and still, with her back well supported, legs uncrossed and feet flat on the floor. Three readings were taken 1 min apart and the mean calculated.

### Pulse wave analysis

The SphygmoCor device (Atcor Medical, West Ryde, Australia) equipped with a hand-held tonometer like a pencil (Millar Instruments, Houston, Texas, USA) was used to perform pulse wave analysis. There is a micromanometer within the tip of the tonometer to record the pressure within the radial artery. The clinic room was temperature controlled and kept quiet and undisturbed. The women were asked to abstain from caffeine, alcohol and smoking, starting from the night before the appointment. Women were asked not to move or speak during the measurements, which were performed in a semi-recumbent position with a left lateral tilt to ensure consistency with the echocardiogram methodology. The radial artery was palpated, and the point of maximal pulsation identified. The tonometer tip was placed at this point. The radial artery waveforms were recorded over 10 s. The aortic pressure waveform is derived from the radial artery waveform by a mathematical transfer function in the Sphygmocor software (Sphygmocor Cardiovascular Management Suite Version 9). Similarly, the software was used to derive the aortic pressure and augmentation index (a measure of arterial stiffness and wave reflection). Good reliability was achieved after a period of supervised training, prior to acquiring measurements for the study.

### Statistical analysis

Baseline characteristics were tested for normality using the Shapiro-Wilk test. Normally distributed data are expressed as mean and standard deviation (SD). Non-normal continuous data are expressed as median and interquartile range). Categorical data are expressed as number and percentage.

Cross-sectional data were subjected to one-way analysis of variance (ANOVA) or Kruskal–Wallis test. Post hoc testing was performed to account for comparisons between the three groups, using Tukey’s test of pairwise comparisons for normally distributed and the Dunn–Bonferroni method for non-normally distributed data respectively. Categorical data are compared using Fisher’s exact test for two groups and the chi-squared test with appropriate degrees of freedom for three groups. A 2-tailed *P*-value of <0.05 was considered statistically significant. Analysis was performed using Stata^®^ (StataCorp. 2015. *Stata Statistical Software: Release 14*. College Station, TX: StataCorp LP).

### Ethical considerations

This research was approved by the Research and Development Department of Sandwell and West Birmingham Hospitals NHS Trust (Reference 13CARD65, 27/11/13), following review by the institution’s Ethics Committee. Prospective approval for the study was also obtained from the Local Research Ethics Committee for West Midlands (Reference 13/WM/0472, 07/01/14). All participants gave written informed consent and confirmed ongoing consent at each follow up appointment. The study was conducted in accordance with the Declaration of Helsinki.

## Results

A total of 115 women were enrolled in the study. The study groups were well matched for age (*P* = 0.74) and ethnicity (*P* = 0.33) with a slight majority (50.4%) coming from non-white racial groups (Table 1). There was no difference in maternal medical history apart from gestational hypertensive disease, which by design is unique to Group 1. Where asthma is noted, the cases were mild. The difference in parity between the groups is also a feature of the study design, since women in Group 1 are required to have a previous pregnancy. The number of women taking aspirin was significantly higher in Group 1, since this daily medication is recommended to women previously affected by hypertension in pregnancy for secondary prevention.

**Table 1:** Demographic and clinical characteristics.

Characteristic	Group 1 Previous hypertension (*n* = 25)	Group 2 Previous normotensive (*n* = 50)	Group 3 Non-pregnant (*n* = 40)	*P*
Age, years	30 (27–33)	29 (25–33)	28 (25–34)	0.74
Parity, *n* (%)				
Nulliparous	0 (0)	22 (44)	28 (70)	<0.001
Parous	25 (100)*^†^	28 (56)	12 (30)	
Ethnicity, *n* (%)				
White	13 (52)	21 (42)	23 (58)	0.49
South Asian	9 (36)	19 (38)	9 (22)	
Black	1 (4)	7 (14)	5 (13)	
East Asian	0 (0)	1 (2)	2 (5)	
Other/mixed	2 (8)	2 (4)	1 (2)	
White	13 (52)	21 (42)	23 (58)	0.33
Non-white	12 (48)	29 (58)	17 (42)	
Medical history, *n* (%)				
Asthma	3 (12)	2 (4)	3 (8)	0.36
Diabetes	1 (4)	3 (6)	0 (0)	0.30
Medications, *n* (%)				
Aspirin	11 (44)^*†^	1 (2)	0 (0)	<0001
Hormonal contraception	0 (0)^†^	0 (0)^‡^	13 (33)	<0.001
Antidepressant	0 (0)	0 (0)	1(3)	0.39
Azathioprine	1 (4)	1 (2)	0 (0)	0.48
Enoxaparin	0 (0)	1 (2)	0 (0)	0.52
Metformin	0 (0)	2 (4)	0 (0)	0.30
Insulin	0 (0)	1 (2)	0 (0)	0.52
Salbutamol	3 (12)	1 (2)	2 (5)	0.19
Thyroxine	0 (0)	3 (6)	0 (0)	0.14
Family history, *n* (%)				
Essential hypertension	11 (44)	13 (26)	15 (38)	0.25
Pregnancy hypertension	3 (12)	4 (8)	5 (13)	0.77
Smoking, *n* (%)				
Current smoker	2 (8)	2 (4)	1 (2)	0.57
Never smoked	17 (68)	43 (86)	38 (95)	0.01

Continuous data are expressed as median (interquartile range). Categorical data are expressed as *n* (%). Post hoc testing: *P < 0.01 Group 1 vs Group 2; ^†^*P* < 0.01 Group 1 vs Group 3; ^‡^*P* < 0.01 Group 2 vs Group 3.

There was no difference in family history of hypertension or current smoking status, however previous smoking habits varied, with the pregnant women previously affected by hypertension more likely to have smoked in the past (*P* = 0.03 for Group 1 vs Group 3). Group 1 comprised 9 women with previous GH, 9 with previous early onset PE and 7 with previous late onset PE. All had normal BP and were free of antihypertensive medication at enrolment.

The pregnant participants were studied at 13 ± 1 completed weeks of gestation. There was no significant difference in weight (*P* = 0.55) or height (*P* = 0.054), although the non-pregnant women tended to be taller. The groups were matched for body mass index (BMI) (*P* = 0.12) and body surface area (BSA) (*P* = 0.74) (Table 2).

**Table 2:** Clinical measurements.

Characteristic	Group 1 Previous hypertension (*n* = 25)	Group 2 Previous normotensive (*n* = 50)	Group 3 Non-pregnant controls (*n* = 40)	*P*
Gestation (weeks)	13 ± 1	13 ± 1	n/a	0.42
Height (m)	1.61 ± 0.08	1.64 ± 0.07	1.66 ± 0.08	0.054
Weight (kg)	73 (58–85)	72 (59–81)	67 (58–83)	0.55
BMI (kg/m^2^)	27.7 (23.2–31.2)	26.5 (22.3–31.6)	23.8 (21.0–28.8)	0.12
BSA (m^2^)	1.78 ± 0.29	1.80 ± 0.22	1.76 ± 0.21	0.74
SBP (mmHg)	115 (113–121)^*†^	108 (100–114)	112 (101–119)	0.001
DBP (mmHg)	71 (65–75)^*^	64 (50–69)	66 (60–72)	0.003
MAP (mmHg)	87 (81–91)^*^	77 (72–82)	81 (74–86)	<0.001
Heart rate (bpm)	79 ± 10^†^	74 ± 9 ^‡^	65 ± 10	<0.001
Hb (g/L)	120 ± 11^†^	122 ± 11^‡^	132 ± 11	<0.001
WCC (×10^9^/L)	9.4 (7.2–10.9)^†^	8.3 (7.0–9.4)^‡^	6.3 (5.3–6.9)	<0.001
Platelets (×10^9^/L)	253 (210–291)	247 (202–292)	270 (249–303)	0.20
Creatinine (µmol/L)	51 (49–54)^†^	53 (50–56)^‡^	66 (62–71)	<0.001

Data expressed as median (interquartile range) or as mean ± standard deviation.

*BMI* body mass index, *bpm* beats per minute, *BSA* body surface area, *DBP* diastolic blood pressure, *Hb* haemoglobin, *MAP* mean arterial pressure, *n/a* not applicable, *SBP* systolic blood pressure, *WCC* white cell count.

Post hoc testing:

^*^p < 0.01 Group 1 vs Group 2.

^†^*p* < 0.01 Group 1 vs Group 3.

^‡^*p* < 0.01 Group 2 vs Group.

The protocol mandated that BP be normal for all women at the start of the study. Accordingly, systolic BP (SBP) was less than 140 mmHg and DBP was less than 90 mmHg for all. Women in Group 1 had significantly higher peripheral SBP at baseline compared to Groups 2 and 3 (*P* < 0.01 for both). DBP and mean arterial pressure (MAP) and central SBP were significantly higher in Group 1 compared to Group 2. Group 2 demonstrated a non-significant reduction in BP compared to Group 3. Heart rate was increased in pregnant women compared to non-pregnant controls (*P* < 0.001).

### Haemodynamics

Whilst the median stroke volume was the same for both groups of pregnant women, the increase compared to non-pregnant controls reached statistical significance only for Group 2 (*P* = 0.003), which persisted after adjusting for BSA (see Table 3). Cardiac output and cardiac index were significantly increased in pregnant women (*P* < 0.001), with similar values observed regardless of previous hypertension. This higher cardiac output was observed alongside reduced resistance, with total vascular resistance index significantly lower in Group 1 (*P* = 0.001) and Group 2 (*P* < 0.001) compared with Group 3. There was no significant difference in the systolic tissue Doppler average velocity at the septal and lateral mitral valve annuli.

**Table 3:** Echocardiographic parameters.

Parameter	Group 1 Previous hypertension (*n* = 25)	Group 2 Previous normotensive (*n* = 50)	Group 3 Non-pregnant controls (*n* = 40)	*P*
Haemodynamics				
Stroke volume (ml)	68 (59–72)	68 (58–76)^‡^	59 (51–69)	0.019
Stroke volume index (ml/m^2^)	37.3 (34.0–42.8)	37.6 (33.6–43.6)^‡^	32.8 (30.0–37.3)	0.017
Cardiac output (L/min)	5.0 (4.6–5.8)^†^	4.8 (4.5–5.6)^‡^	3.8 (3.1–4.5)	<0.001
Cardiac index (L/min/m^2^)	3.0 (2.7–3.2)^†^	2.8 (2.4–3.3)^‡^	2.1 (1.8–2.4)	<0.001
Total vascular resistance (dyne.s/cm^5^)	1.4 (1.2–1.5)^†^	1.2 (1.1–1.5)^‡^	1.7 (1.5–2.1)	<0.001
Total vascular resistance index (dyne.s/cm^5^/m^2^)	0.80 (0.65–0.89)^†^	0.69 (0.58–0.89)^‡^	0.97 (0.83–1.20)	<0.001
Average s’ (cm/s)	9.6 (8.5–10.2)	9.5 (8.8–10.1)	9.5 (8.9–10.4)	0.84
Structure				
Left atrium volume (ml)	41 (31–45)	39 (31–46)	42 (30–51)	0.71
Left atrium volume index (ml/m^2^)	22 (18–28)	22 (17–26)	23 (18–28)	0.76
Left ventricle volume (ml)	98 (84–127)^†^	110 (91–121)^‡^	91 (80–101)	<0.001
Left ventricle volume index (ml/m^2^)	59 (52–64)^†^	60 (50–71)^‡^	51 (44–58)	<0.001
Left ventricular mass (g)	117 (95–152)	119 (97–143)	103 (81–129)	0.10
Left ventricular mass index (g/m^2^)	70.9 (57.4–82.5)^†^	65.4 (56.8–78.7)	61.6 (49.7–69.2)	0.03
Left ventricular end diastolic dimension (cm)	4.5 (4.3–5.0)	4.5 (4.2–4.8)	4.4 (4.3–4.8)	0.93
Posterior wall thickness at end diastole (cm)	0.9 (0.7–1.1)	0.9 (0.8–1.0)^‡^	0.8 (0.7–0.9)	0.047
Interventricular septum thickness at end diastole (cm)	0.79 (0.69–0.91)	0.73 (0.66–0.87)	0.72 (0.57–0.82)	0.10
Relative wall thickness	0.38 (0.32–0.49)	0.40 (0.33–0.46)	0.36 (0.29–0.41)	0.11
Diastolic function (mitral inflow)				
Early filling (E) (cm/s)	85 (75–99)^†^	88 (77–96)^‡^	74 (67–85)	<0.001
Atrial filling (A) (cm/s)	62 (51–67)^*†^	48 (44–62)	49 (40–74)	0.005
E/A ratio	1.40 (1.15–1.64)^*†^	1.79 (1.55–2.25)	2.03 (1.54–2.32)	<0.001
e’ (cm/s)				
Septal	11.2 (9.7–12.5)	11.2 (9.9–12.9)	11.6 (10.2–12.7)	0.90
Lateral	14.0 (12.6–16.2)	15.4 (13.5–18.3)	15.7 (14.7–17.7)	0.17
E/e’ (average from septal and lateral)	6.6 (5.7–8.2)^†^	6.5 (5.2–7.5)^‡^	5.4 (4.6–6.4)	0.001
Derived values including elastance				
Left ventricular ejection fraction	0.63 (0.59–0.67)	0.63 (0.60–0.67)	0.66 (0.62–0.69)	0.066
Left ventricular end diastolic pressure (mmHg)	10.1 (9.0–12.1)^†^	9.9 (8.4–11.2)^‡^	8.6 (7.9–9.9)	0.008
Arterial elastance (Ea)	1.6 (1.4–1.8)	1.4 (1.2–1.7)^‡^	1.7 (1.5–1.9)	0.005
Arterial elastance index (EaI)	0.95 (0.79–1.11)	0.79 (0.72–0.95)^‡^	0.95 (0.80–1.10)	0.004
End-systolic elastance (Ees)	2.0 (1.8–2.3)	1.9 (1.6–2.2)^‡^	2.3 (1.8–2.7)	0.015
End-diastolic elastance (Eed)	0.9 (0.08–0.11)	0.08 (0.07–0.10)	0.08 (0.07–0.10)	0.24
Arterial-ventricular interaction (Ea/Ees)	0.85 (0.78–0.88)	0.81 (0.74–0.90)	0.76 (0.71–0.85)	0.084
Arterial-ventricular interaction index (EaI/Ees)	0.47 (0.42–0.59)	0.44 (0.40–0.53)	0.45 (0.41–0.48)	0.22

Data expressed as median (interquartile range) or as mean ± standard deviation.

*E* mitral valve early filling on Pulsed-Wave Doppler, *e’* velocity of early myocardial relaxation measured on tissue Doppler imaging, *A* mitral valve late (atrial) filling.

Post hoc testing:

*p < 0.01 Group 1 vs Group 2.

^†^*p* < 0.01 Group 1 vs Group 3.

^‡^*p* < 0.01 Group 2 vs Group.

### Cardiac structure

The size of the left atrium was unchanged across groups (*P* > 0.05). Pregnant women exhibited a significant increase in LV volume and its index to BSA (*P* < 0.001). Women with previous GH had a significantly higher LV mass index compared to non-pregnant women (*P* = 0.006), whilst the increase compared to pregnant women without hypertension history was not significant after Bonferroni adjustment. There was no significant difference in LV end diastolic dimension, interventricular septum thickness or relative wall thickness (*P* > 0.05). The difference in posterior wall thickness was not significantly different after post hoc testing.

### Diastolic function

Significant differences were observed for mitral inflow velocities. The maximum early mitral valve inflow velocity on Pulsed-Wave Doppler (E wave) was significantly greater in the pregnant women irrespective of former hypertension history (*P* = 0.003 Group 1 vs Group 3; *P* < 0.001 Group 2 vs Group 3). The maximum late diastolic mitral inflow velocity was significantly increased in the women with previous hypertension, compared to both unaffected pregnant women and non-pregnant women (*P* = 0.001 for both comparisons). Similarly, the calculated E/A ratio of early to late ventricular filling in diastole, was significantly lower when pregnancy was complicated by previous gestational hypertensive disease (*P* < 0.001). There was no difference in e’, the velocity of early myocardial relaxation measured on tissue Doppler imaging, between the three groups (*P* > 0.05). The E/e’ ratio of early mitral inflow to the average tissue Doppler early myocardial relaxation at the septal and lateral mitral valve annuli, was significantly increased in both groups of pregnant women compared to the non-pregnant controls (*P* = 0.001 for both).

### Elastance parameters and other derived values

Left ventricular ejection fraction (LVEF) was similar in all groups (*P* > 0.05). LV end diastolic pressures were higher in each group of pregnant women compared to non-pregnant controls but not to each other (*P* = 0.008). There was a significant decrease in arterial elastance index (EaI, *P* < 0.001) and LV end-systolic elastance (Ees, *P* = 0.002) in healthy pregnant women in Group 2 compared to non-pregnant controls. Similar values of EaI were seen in Groups 1 and 3 and the reduction in Ees in Group 1 was not statistically significant after post hoc testing. There was no difference in EaI/Ees, the arterial-ventricular interaction index, between the three groups (*P* > 0.05).

### Arterial stiffness

Augmentation index, standardised to heart rate 75 beats per minute, was significantly increased in Group 1 with previous hypertension compared to Group 2 (*P* = 0.004) and to Group 3 (*P* < 0.001, Table 4). There was no significant difference in arterial stiffness in pregnancy without hypertension history compared to non-pregnant women (*P* > 0.05).

**Table 4:** Pulse wave analysis.

Parameter	Group 1 Previous hypertension (*n* = 25)	Group 2 Previous normotensive (*n* = 50)	Group 3 Non-pregnant controls (*n* = 40)	*P*
Augmentation index	15 (6–19)	5 (−2 to 12)	7 (2–16)	0.06
Augmentation index HR75	16 (4–21)*^†^	4 (−3 to 14)	2 (−6 to 11)	0.003
Augmentation index HR75 adjusted for MAP	0.16 (0.05–0.24)*^†^	0.05 (−0.03 to 0.17)	0.03 (−0.08 to 0.13)	0.003

Data expressed as median (interquartile range) or as mean ± standard deviation. HR75 denotes adjustment to standardise for heart rate 75 beats per minute; *MAP* mean arterial pressure. Post hoc testing: ^*^p < 0.01 Group 1 vs Group 2; ^†^*p* < 0.01 Group 1 vs Group 3.

## Discussion

The SBP in Group 1 at 13 ± 1 weeks of gestation was significantly higher than both other groups after correcting for multiple comparisons (*P* < 0.001 vs Group 2; *P* = 0.007 vs Group 3). This observation was also reported in a 2-year follow up study of women after preeclamptic pregnancy [12]. The concept of prehypertension was formally introduced into American guidelines over a decade ago [25]. Patients with a SBP of 120–139 mmHg or a DBP of 80–90 mmHg are classified as prehypertensive. European guidelines [26, 27] designate this group ‘high normal’. Some 5/50 women in Group 2 and 10/25 women in Group 1 would have been classed as prehypertensive according to the Joint National Committee Guideline [25]. The proportion of women with prehypertension in Group 1 is significantly higher (*P* = 0.002). People with prehypertension have double the odds of developing hypertension compared to those with lower BP [25]. When observed at postpartum follow up over 4–10 years after PE, prehypertension is associated with asymptomatic heart failure [28]. Over time, prehypertension causes LV hypertrophy and diastolic dysfunction [29]. Previous PE, especially in association with prehypertension, is independently associated with an increased risk of subclinical cardiac failure [28]. The relationship between BP and risk of cardiovascular disease events is continuous and independent of other risk factors. If prehypertension is identified (rather than simply stating that BP is in the “normal” range), primary prevention strategies, starting with lifestyle modification, can be implemented.

The increase in stroke volume index was only significant in the group of pregnant women unaffected by previous hypertension. Previous studies have reported a wide range of values for stroke volume in pregnancy, with considerable disagreement regarding the expected physiological, longitudinal changes [30]. Stroke volume is affected by extracardiac factors (preload and afterload). Arterial BP and vascular tone create the afterload against which the ventricles must eject blood. If afterload is increased then the stroke volume will decrease. The interplay between the pressure and volume components means that the reduction in stroke volume with an increased afterload depends on the end diastolic volume, and whether a secondary increase in preload can result in a greater contractile force according to the Frank-Starling principle.

The physiological increase in LV volume and LV mass corresponded with the increase in BSA and hence did not reach statistical significance after indexation. A large component of the increased body weight throughout pregnancy is the increasing size of the feto-placental unit. Therefore, if the left ventricle increases in size relative to placenta and its demands for blood supply, it makes mathematical sense that indexation would ‘cancel out’ any significance in the longitudinal comparison of LV structure.

The increase in LV mass compared to non-pregnant women was only significant for the women previously affected by hypertension. This increase in LV mass in Group 1 occurred in conjunction with increased cardiac output. In a systematic review of echocardiographic structure and function in HDP [14] and in a review of cardiac function in normal pregnancy by Melchiorre et al. [30], cardiac output was a parameter with considerable variation in reported measures and trends. This is likely to be due to different patient characteristics, timing of assessment and methodological variation in relation to measurement of stroke volume. It is also possible that the legacy of a hypertensive disorder in pregnancy is different according to its association with fetal growth restriction. A recent study demonstrated that PE is associated with increased cardiac output, but when fetal growth restriction coexists, cardiac output is reduced and peripheral vascular resistance is increased [31]. It remains to be shown whether the cardiovascular profile is altered due to maladaptation in the index pregnancy, or whether there was pre-existing pathology in the cardiovascular system unmasked by pregnancy.

A recent meta-analysis showed that LVM and RWT both increase in normal pregnancies, which demonstrates concentric rather than eccentric hypertrophy [32]. Eccentric hypertrophy is seen in healthy athletes in response to training. Physiological remodelling is seen in healthy pregnancy, as a woman develops a heart akin to a sportswoman. This type of hypertrophy was previously thought to be a sign of pathology in hypertensive pregnancies [33]. When there is increased volume load, the end diastolic pressure increases. The cardiac myocytes increase so the wall thickness increases. This compensates for the increase in pressure.

A greater increase in LVM and RWT was demonstrated in hypertensive pregnancies. This shows that in some cases the pregnant heart reaches its limit of physiological adaptation (where the remodelling would be expected to be eccentric) and the hypertrophy becomes the kind more associated with pathology. This maladaptation to chronic volume overload even in healthy pregnancy has recently been reported in an echocardiographic study [34]. It has been shown that some 40% of women with PE have persistently abnormal cardiac structure and function up to 1 year postpartum, with diastolic dysfunction amounting to subclinical heart failure [12].

The increased early diastolic mitral inflow (E wave) in pregnancy is typical of healthy, fit individuals. As the ventricular wall recoils there is negative pressure in the ventricle, so blood is sucked down the pressure gradient from the atrium across the mitral valve. This results in a higher E wave. There is relatively little atrial filling as most of the volume has already flowed out of the atrium, leaving a smaller atrial contribution. This is the pattern seen in healthy pregnancy and in the non-pregnant controls. Since there is an increased volume load in pregnancy, the already stretched heart is less compliant. This explains the reduction in E/A seen in normal pregnancy. In the women affected by previous hypertension, late diastolic filling caused by atrial contraction was greater, leading to a significantly lower E/A ratio in this group. The larger A wave usually reflects compensation for reduced early filling, after its being impaired by a stiffer ventricle. In the case of Group 1, the results suggest a trend towards a ‘pseudonormal’ filling pattern which is a marker of diastolic dysfunction. As the left atrial pressure rises as a marker of progressive diastolic dysfunction, an increase in the E wave is caused not by reduced pressure in the ventricle, but rather by increased pressure from the atrium driving blood through the mitral valve in early diastole and in this pattern both E and A are increased as seen in Group 1. The E and A waves are affected by the loading conditions of pregnancy [35, 36]. Pregnancy affects volume haemostasis with an ~1600 ml increase in the intravascular compartment (1300 ml extra plasma volume and 30 ml extra red blood cell volume) [37].

In the healthy pregnant women arterial elastance index was significantly lower, than in women with prior hypertension. Arterial elastance relates to the ability of the aorta to receive blood. In healthy pregnancy there is increased compliance in the arterial system. In our study the women with prior hypertension did not demonstrate this physiological adaptation. Both peripheral and aortic arterial stiffness has been demonstrated in PE [38]. Increased arterial stiffness has been shown to be present postpartum [39, 40]. To our knowledge this is the first demonstration of increased arterial stiffness in a normotensive pregnancy following gestational hypertensive disease. This is consistent with other studies showing increased arterial stiffness after pregnancy hypertension [41, 42]. This adverse effect leads to increased risk of future hypertension, coronary artery disease and heart failure [43].

There were some notable limitations. Firstly, indexing in pregnant women may be misleading because the change in the body contour is not consistent with the standard way in which BSA is calculated [44]. Furthermore it is debatable whether pre-pregnancy or baseline body weight should be used in the calculations or whether the actual weight at the time of assessment should be incorporated [44]. Scaling according to maternal size is a crude correction, since it is not possible to correct for the metabolic demands of pregnancy. There are various approaches to adjusting for maternal height and weight for example using height only [45], BSA [46] or no quantitative adjustment for maternal anthropometry [47]. We have used indexing for specific pre-specified derived measurements, and in each case have displayed the raw data alongside the adjusted data. The confounding effect of body size in cardiovascular medicine and in particular in pregnancy is important, and it would be useful to establish a consensus on how indices should be reported. Using BSA as an indicator for TVR has been challenged in the literature and attention is drawn to the relationship between vascular resistance and aging [48]. No adjustment for age was made in this study given that all subjects belonged to a relatively narrow reproductive age. Furthermore, adjustment of augmentation index to heart rate of 75 beats per minute is not universally accepted. This method is recommended by the manufacturers of SphygmoCor^®^, and has been used in previous studies in pregnancy [49–51].

Thirdly, the pregnant heart is more spherical compared to the more ellipsoid non-pregnant heart. There is no geometrical assumption in the Simpson method of discs to calculate the LV volume. This method was employed to calculate LVEF. Fourthly, Simpson’s method represents the longitudinal contractile function only and does not account for the radial contractile function. Additionally, speckle tracking technology (which is more independent of the loading conditions) can be used to assess complex torsional heart movement in order to detect preclinical impairment of LV function [52]. Speckle tracking was not available during recruitment for this study.

Finally, this study lacks data from before conception and the earliest weeks of pregnancy when haemodynamic changes begin. Preconceptual data, although methodologically challenging, can determine a woman’s baseline cardiac function, rather than using non-pregnant or postpartum indices as a control. Future study designs should seek to incorporate the preconceptual period.

## Conclusion

We have described cardiovascular system differences in pregnancy, depending on history of GH or PE. We have shown increased prevalence of prehypertension, and increased arterial stiffness in pregnant women previously affected by gestational hypertensive disease. An increased atrial component to ventricular filling reflects altered diastolic function after hypertensive pregnancy.

### Summary table

#### What is known about topic

- Hypertensive disorders of pregnancy are an important cause of morbidity and mortality, impacting on both maternal and fetal wellbeing, but mechanisms implicated are not well understood.
- There is a significant increase in arterial stiffness indices in women with preeclampsia compared to women with gestational hypertension and normotensive pregnant women.

#### What this study adds

- Pregnant women with previous hypertension in pregnancy have features of diastolic dysfunction manifested by increased late diastolic transmitral flow velocities.
- Pregnant women without prior hypertension demonstrated adoptive changes in arterial compliance, which is not seen in pregnancy with prior hypertension, where increased arterial stiffness was observed.
- Women with previous gestational hypertension are at increased future cardiovascular risk due to altered cardiac and vascular function and require effective risk mitigation.
